# Enhancing generosity through movement: association between physical exercise and charitable donation behavior

**DOI:** 10.3389/fpsyg.2025.1606795

**Published:** 2025-07-22

**Authors:** Xiaojuan Yang

**Affiliations:** ^1^School of Management, Zhejiang University of Technology, Hangzhou, China; ^2^School of Economics and Trade, Anhui Technical College of Mechanical and Electrical Engineering, Wuhu, China

**Keywords:** physical exercise, sense of social responsibility, subjective well-being, donation participation, donation amount

## Abstract

**Introduction:**

This study explores the relationship between physical exercise and charitable donation behavior in the context of China’s pursuit of common prosperity. Specifically, it examines whether individuals who regularly engage in physical activity are more likely to donate to charitable causes and in greater amounts, as well as the psychological mechanisms underlying this relationship.

**Methods:**

Using nationally representative data from the Chinese General Social Survey (CGSS2012), we conducted multivariate regression analyses to estimate the association between physical exercise and donation behavior. To ensure the robustness of the results, we performed a series of sensitivity analyses, including propensity score matching, alternative outcome measures, and different model specifications. Mediation analyses were also conducted to test the potential mediating roles of social responsibility and subjective well-being, drawing on theories of altruism and reciprocity.

**Results:**

The findings indicate a significant positive association between participation in physical exercise and both the likelihood of charitable donation and the amount donated. Robustness checks confirmed the stability of these results. Furthermore, heterogeneity analyses revealed that this relationship does not vary significantly across gender, age, or household registration status. Mediation analysis showed that both social responsibility and subjective well-being partially mediate the relationship between exercise and donation behavior.

**Discussion:**

The findings of this study underscore the potential societal benefits of promoting mass participation in physical exercise. By fostering a sense of social responsibility and enhancing subjective well-being, physical exercise may serve as a pathway to broader civic engagement. Promoting mass sports participation could thus support societal goals such as advancing common prosperity in China.

## 1 Introduction

Common prosperity is a defining characteristic of Chinese modernization. Charitable activities play a vital role in regulating income distribution, narrowing the wealth gap, and maintaining social equity and stability, making them an important pathway to achieving the goal of common prosperity. The report of the Party’s 20th National Congress proposed to “build a coordinated system of primary distribution, redistribution and third distribution,” and required “to guide and support enterprises, social organizations and individuals with willingness and ability to actively participate in public welfare and philanthropy,” which provided a fundamental guideline for the development of China’s philanthropy.

According to the Charity White Paper: China’s Charity Development Report (Yang and Zhu, 2024), the overall scale of charitable resources in China has continued to expand in recent years. However, individual donations have consistently accounted for a relatively small proportion of total social donations, which is a primary reason why China’s overall charitable giving lags behind that of developed countries (Luo et al., 2019). Identifying effective and sustainable ways to promote individual charitable donations among Chinese people is therefore a critical issue in the process of realizing common prosperity.

As a social activity, physical exercise has significant social functions. Numerous studies have explored the beneficial impacts of physical exercise on physical and mental health (Fossati et al., 2021), life satisfaction (Zhang et al., 2022), and happiness (Zhang and Chen, 2019). In particular, physical exercise has been consistently associated with improved subjective well-being (Buecker et al., 2021). The physiological mechanisms through which physical exercise enhances subjective well-being include the release of endorphins, increased levels of dopamine and serotonin, and reduction of cortisol (Paluska and Schwenk, 2000; Mathew and Paulose, 2011; Cheng et al., 2025). However, research on how physical exercise contributes to philanthropic activities relatively limited. Against the backdrop of advancing common prosperity alongside the “Sports Power” strategy, does physical exercise promote individuals’ charitable donation behavior in China? What are the potential transmission mechanisms behind this effect? This study, based on data from Chinese General Social Survey 2012 (CGSS2012), uses a multivariate regression model to empirically examine the impact of physical exercise on charitable donation behavior. It further explores the potential pathways through which physical exercise influences charitable donations.

## 2 Literature review and research hypothesis

### 2.1 Physical exercise and charitable donation behavior

Physical exercise is a form of physical activity that combines natural forces and health practices, aimed at enhancing physical fitness, regulating mental state, and enriching cultural life (Yao et al., 2023). It has a significant positive impact on various aspects of an individual’s cognition, emotions, willpower, and behavior. Through physical activities, individuals not only improve their physical and mental health but also enhance social cognition, learn and follow social norms, and thereby improve their ability to adapt to society (Dilnoza, 2023). Physical exercise can also promote the development of positive psychological qualities, such as perceiving social support and improving psychological capital, which in turn positively impacts prosocial behavior (Lu et al., 2022).

Previous literature has focused on specific groups, such as athletes and students, to explore the impact of physical exercise on prosocial behavior, revealing that the mechanisms of influence differ across these groups. Kavussanu (2006) was the first to introduce the concept of prosocial and antisocial behavior into the field of sports, highlighting the role of prosocial behavior in sports. O'donnell and Barber (2018) conducted experimental studies that indicated children who participate in physical exercise exhibit significant improvements in emotional regulation and interpersonal relationship-building skills, which subsequently foster prosocial behavior. Adolescents who experience stressful events can stimulate empathy and post-traumatic growth through physical exercise, further promoting prosocial behavior (Wang et al., 2023). Charitable donations, as a voluntary act of giving love and helping the disadvantaged, are also a form of prosocial behavior (Clavien and Chapuisat, 2013). Based on the above analysis, this study proposes the following hypothesis:

*H1*: Physical exercise is positively associated with individuals’ charitable donation behavior.

### 2.2 Mechanism analysis

Individuals’ donation behavior may be driven by pure altruism (Carlson and Zaki, 2022) or reciprocal motives, such as seeking internal psychological rewards (Andreoni, 1990). Altruism refers to a psychological motivation that focuses on the interests of others without considering one’s own interests. It is a voluntary and clearly dedicated motivation aimed at helping others (Khalil, 2004). One of the main manifestations of an individual’s altruistic motivation is a sense of social responsibility, which is a tendency to voluntarily pursue the common good of society and its members (Starrett, 1996). Social responsibility is more of an internal social value; individuals with a strong sense of social responsibility believe that they have a duty to contribute to society. Therefore, social responsibility drives individuals to engage in prosocial behavior (Berkowitz and Daniels, 1963). Research has shown that individuals with a higher sense of social responsibility tend to donate blood more frequently (Steele et al., 2008).

Physical exercise is an effective means of promoting the formation of individual values and a sense of responsibility. Previous studies have verified the positive role of sports education in shaping an individual’s social responsibility (Walsh, 2007). Sports typically have clear rules, and participants must adhere to these rules. This rule-consciousness can extend into social life, encouraging people to better follow social norms and enhancing their sense of social responsibility. Physical exercise enhances individuals’ self-efficacy, nurtures a positive and optimistic outlook on life, strengthens psychological resilience, and cultivates valuable psychological capital (Lu et al., 2022), which plays a crucial role in shaping a strong sense of social responsibility (Maharani et al., 2025). Consequently, engaging in physical exercise can foster a greater sense of social responsibility, thereby promoting altruistic behaviors such as charitable donations. Based on the above analysis, we propose the following hypothesis:

*H2*: Social responsibility plays a mediating role in the positive relationship between physical exercise and charitable donation behavior.

According to the warm glow theory, people’s act of giving is not merely out of concern for the welfare of others, but may also be mixed with expectations of personal spiritual rewards (Andreoni, 1990). Participating in physical exercise is a meaningful activity that can induce subjective well-being (Tkach and Lyubomirsky, 2006). Kushlev et al. (2022) using large-scale survey data from 163 countries revealed happy people give more of their time and money to others. Subjective well-being refers to individuals’ subjective evaluations of their quality of life based on self-defined standards, and it is an important indicator of an individual’s life quality. Wiese et al. (2018) suggested that the impact of physical exercise on subjective well-being is primarily reflected in life satisfaction and positive emotions. Based on the analysis of Maslow’s hierarchy of needs theory, Zhu (2020) pointed out that individuals with high life satisfaction not only have the ability to donate to charity, but also the motivation to donate to charity. Such people are more inclined to pursue spiritual value needs and desire to gain social reputation and respect from others. Charitable donation provides them with a way to realize these needs. Emotion forms the foundation of helping behavior. Positive emotions typically refer to pleasant emotional experiences, such as happiness, joy, excitement, enthusiasm, and contentment (Pressman et al., 2019). According to the mood maintenance theory, positive emotions can promote prosocial behavior, as people engage in prosocial actions to maintain their existing positive emotions (Carlson et al., 1988). Experimental studies (Isen et al., 1976) and field studies (George and Brief, 1992) have shown that inducing positive emotions increases helping behavior, which is consistent with the “Feeling good-doing good” effect (Rosenhan et al., 1981). Based on the above analysis, the following hypothesis is proposed:

*H3*: Subjective well-being plays a mediating role in the positive relationship between physical exercise and charitable donation behavior.

**Figure 1:**
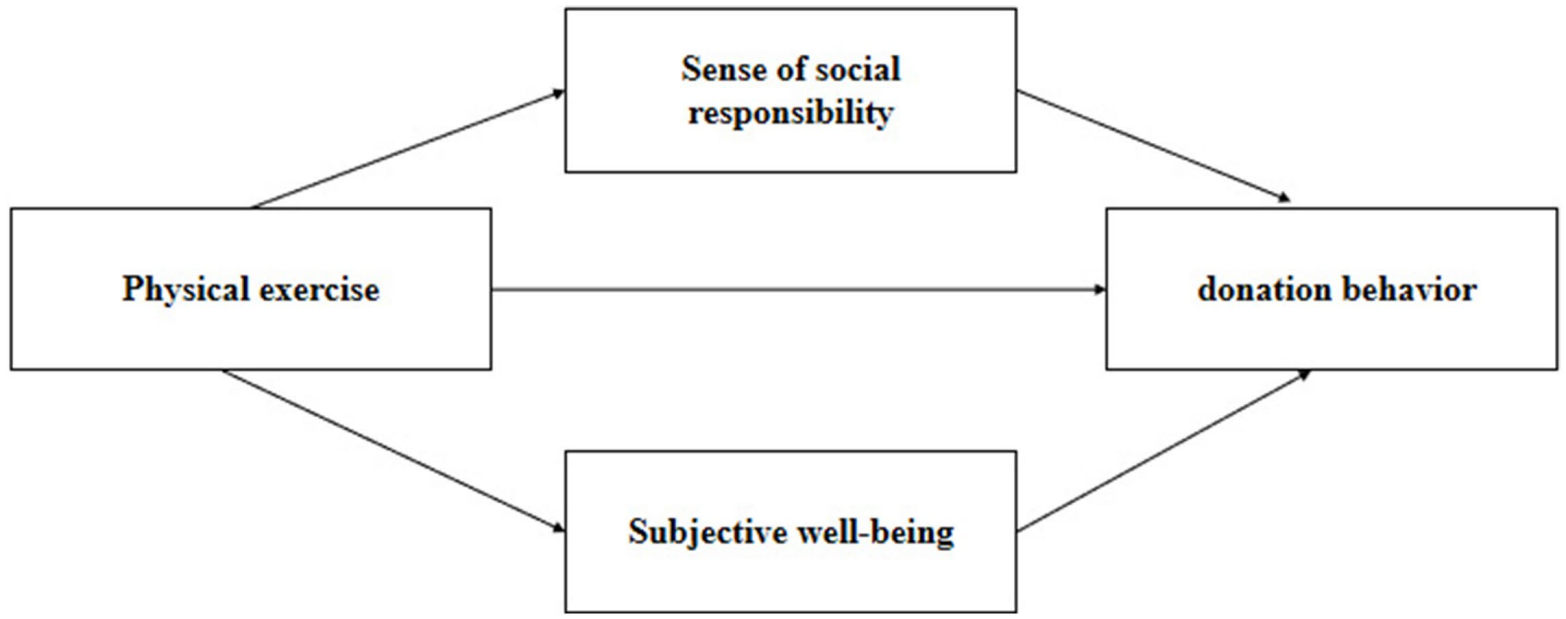
Theoretical frame.

According to the discussion above, the proposed theoretical framework is illustrated in Figure 1.

## 3 Research methods

### 3.1 Data source

The Chinese General Social Survey (CGSS) is China’s first nationwide, comprehensive, and continuous academic survey project. It systematically gathers data across multiple levels, including society, communities, families, and individuals. Among the available CGSS datasets, the CGSS2012 includes all the core variables essential for this study, making it as the primary data source for this research. The CGSS2012 collected a total of 11,765 valid questionnaires, of which 5,819 were related to the theme of charitable donations. Based on the research requirements, questionnaires with missing or anomalous values in core or control variables were excluded, resulting in a final sample of 5,101 valid samples.

### 3.2 Variable description

The dependent variable is charitable donation behavior, which is divided into donation participation and donation amount. Donation participation is measured using the question in CGSS2012: “In 2011, did you personally make any social donations in the form of money, goods, or property? This refers to voluntary donations made by you personally to individuals or organizations in society without expecting any return.” Responses of “Yes” are coded as 1, and “No” as 0. The donation amount variable is measured through the question: “In 2011, to which of the following categories did you make donations? What was the total amount of your donations to each category, converted into RMB?” The total donation amount for each respondent is calculated by summing their donations across all categories. To reduce data volatility and outliers, the variable is log-transformed.

The independent variable is the frequency of physical exercise. This is assessed based on the CGSS2012 question: “In the past year, have you regularly participated in physical exercise during your free time?” Responses include “Daily,” “Several times a week,” “Several times a month,” “A few times a year or less” and “Never,” with values assigned from 5 to 1, respectively.

Mediator variables include sense of social responsibility and subjective well-being. Referring to Wang et al. (2023), the sense of social responsibility is measured using the CGSS2012 item: “I want to contribute to society.” Respondents rate their level of agreement, with “Strongly Disagree” to “Strongly Agree” coded from 1 to 7. The variable of Subjective Well-Being is measured using the question: “Overall, how happy do you feel with your life?” (Ding et al., 2021). Responses range from “Very unhappy,” “Somewhat unhappy,” “Neutral,” “Somewhat happy” to “Very happy,” coded from 1 to 5.

### 3.3 Control variables

The control variables include respondents’ demographic characteristics such as gender, age, years of education, marital status, ethnicity, household registration, self-rated health status, income, party membership, subjective social class. Given that social trust and social network are key components of social capital and can influence individuals’ giving behavior (Wu et al., 2018), this study incorporates both as control variables in the analysis. Social network is assessed using two items: “Please indicate how often you participate in social and recreational activities with your neighbors (such as visiting each other, watching TV together, having meals, playing cards, etc.)”; “Please indicate how often you participate in similar activities with friends.” Both items provide seven response options, ranging from “almost every day” to “never,” assigned values from 7 to 1, respectively. The mean of these two values is used to construct the social network variable (Du and Wan, 2022) (see Table 1).

**Table 1:** Variable definition and descriptive statistics of variables.

Variable categories	Variable name	Values and meaning	Percentage/mean
Dependent Variable	Donation participation(DP)	Yes = 1, No = 0	32.69%
	Donation amount(DA)	The logarithm of respondents’ total donations in the past year	1.64
Independent Variable	physical exercise(PE)	Daily = 5, Several times a week = 4, Several times a month = 3, A few times a year or less = 2, Never = 1	2.10
Mediating variable	Sense of social responsibility (SSR)	Strongly disagree = 1, Disagree = 2, Neither agree nor disagree = 3, Agree = 4, Strongly agree = 5	5.36
	Subjective well-being (SWB)	Very unhappy = 1, Somewhat unhappy = 2, Neither happy nor unhappy = 3, Somewhat happy = 4, Very happy = 5	3.84
Control variable	Gender	Male = 1, female = 0	51.45%
	Age	The respondent’s actual age at the time of the survey	49.26
	Ethnicity	Han nationality = 1, others = 0	90.96%
	Party membership	Member of the Communist Party of China = 1, else = 0	12.40%
	Household registration	Urban = 1, rural = 0	52.53%
	Marital status	Married = 1, others = 0	83.72%
	Education	No education = 0, Primary school = 6, Junior high school = 9, General high school/Vocational high school/Secondary technical school = 12, Associate degree = 15, Bachelor’s degree and above = 16, Master’s and Doctoral degrees = 19	8.76
	Self-rated health status	Very unhealthy = 1, Somewhat unhealthy = 2, Average = 3, Somewhat healthy = 4, Very healthy = 5	3.52
	Income	The natural logarithm of annual income plus one	8.62
	Subjective social class	Self-perception of social class (1–10), where 1 = lowest class and 10 = highest class	4.17
	Social trust	“In general, most people in this society can be trusted”(1 = “strongly disagree” to 5 = “strongly agree”)	3.49
	Social network	Almost every day = 7; one to two times a week = 6; several times a month = 5; about once a month = 4; several times a year = 3; once a year or less = 2; never = 1	4.18

**Figure 2:**
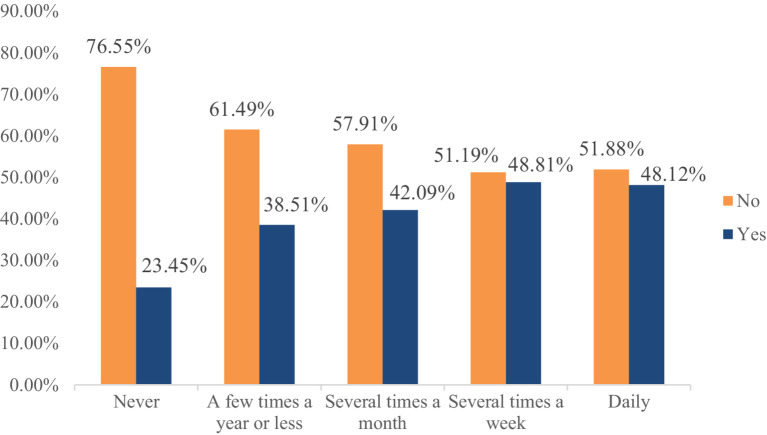
Percentage of donation participation by frequency of physical exercise.

**Figure 3:**
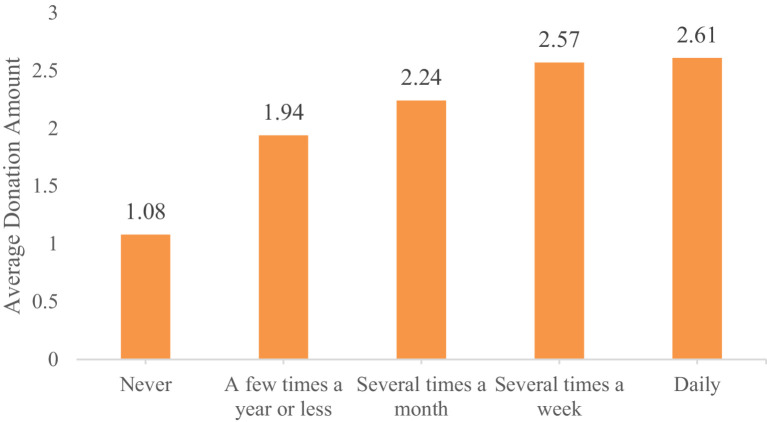
Average donation amount by frequency of physical exercise.

The descriptive statistics show that 32.69% of the respondents participated in charitable donations, reflecting a relatively low level of donation participation among Chinese residents. Specifically, the proportion of residents participating in charitable donations increases with higher frequencies of physical exercise, ranging from 23.45% for those who never exercise to 48.12% for those who exercise daily. The specific proportions are 23.45, 38.51, 42.09, 48.81, and 48.12% for each frequency category, respectively (Figure 2). Similarly, the donation amounts for each group, based on exercise frequency, also show an upward trend, with values of 1.08, 1.94, 2.24, 2.57, and 2.61, respectively (Figure 3). These results preliminary suggest a positive relationship between physical exercise and residents’ donation behavior.

### 3.4 Empirical analysis methods

#### 3.4.1 Baseline regression model

Given the binary nature of the dependent variable donation participation and the continuous nature of donation amount, a Logistic regression model and an OLS regression model are constructed respectively, to examine the impact of physical exercise on charitable donation behavior. The model equations are as follows:


(1) $$\text{Donation}\prime =\alpha_{0}+\alpha_{1}{\text{execise}}_{i}+\alpha_{2}{\text{controls}}_{i}+\varepsilon$$

#### 3.4.2 Mediation effect model

The mediation effect model equations are as follows:


(2) $${\text{Mediator}}_{i}=\beta_{0}+\beta_{1}{\text{execise}}_{i}+\beta_{2}{\text{controls}}_{i}+\theta$$

(3) $$\text{Donation}\prime \prime =\gamma_{0}+\gamma_{1}{\text{execise}}_{i}+\gamma_{2}{\text{Mediator}}_{i}+\gamma_{3}{\text{controls}}_{i}+\delta$$

Through models (2) and (3), the mediating role of sense of responsibility and subjective well-being is tested. “Mediator” refers to the mediating variables, sense of social responsibility and subjective well-being, which explain the mechanism through which physical exercise influences charitable donation behavior.

## 4 Empirical analysis and results

### 4.1 Benchmark regression

Table 2 reports the regression results of the relationship between physical exercise and charitable donation behavior (Equation 1). Model 1 and Model 2 present the effect values for donation participation, while Model 3 and 4 present the effect values for donation amount. The results of Model 1 and 3 indicate that the effect values of physical exercise are 0.31 and 0.42, respectively, both significant at the 1% level. The regression results of Model 2 and 4 show that even after incorporating control variables, physical exercise continues to have a significant positive effect on both donation participation and donation amount, with effect values of 0.20 and 0.25, respectively, both significant at the 1% level. These findings provide evidence supporting hypothesis H1, which suggests that physical exercise positively associates with both donation participation and donation amount.

**Table 2:** Analysis of the relationship between physical exercise and charitable donation behavior.

Variable	Model 1 Logistic	Model 2 Logistic	Model 3 OLS	Model 4 OLS	Model 5 Logistic	Model 6 OLS	Model 7 Probit	Model 8 Tobit
Physical exercise	0.31^***^ (0.02)	0.20^***^ (0.02)	0.42^***^ (0.02)	0.25^***^(0.03)	0.23^***^(0.04)	0.06^***^(0.01)	0.12^***^ (0.01)	0.11^***^ (0.02)
Gender		−0.27^***^ (0.07)		−0.25^***^(0.07)	−0.32^***^(0.11)	−0.08^***^(0.03)	−0.16^**^ (0.04)	0.13^*^ (0.07)
Age		−0.02^***^ (0.00)		−0.01^***^(0.00)	−0.01^***^(0.01)	−0.00(0.02)	−0.01^***^ (0.00)	0.00 (0.00)
Education		0.08^***^ (0.01)		0.08^***^(0.01)	0.09^***^(0.02)	0.01^***^(0.01)	0.04^***^ (0.01)	0.05^***^ (0.01)
Marital status		0.24^***^ (0.08)		0.21^***^(0.08)	−0.14(0.14)	−0.02(0.03)	0.13^***^ (0.05)	0.28^***^ (0.09)
Party membership		0.47^***^ (0.10)		0.76^***^(0.11)	0.75^***^(0.14)	0.26^***^(0.04)	0.30^***^ (0.06)	0.29^***^ (0.09)
Ethnicity		−0.42^***^ (0.11)		−0.49^***^(0.11)	−0.34^*^(0.18)	−0.09^**^(0.04)	−0.25^*^ (0.07)	−0.16 (0.11)
Household registration		0.29^***^ (0.08)		0.35^***^(0.08)	−0.04(0.06)	−0.02(0.03)	0.17 (0.05)	0.16^*^ (0.08)
Self-rated health status		−0.01 (0.03)		−0.01(0.03)	−0.06(0.06)	−0.01(0.01)	0.00 (0.04)	−0.01 (0.04)
Income		0.04^***^ (0.01)		0.05^***^(0.01)	0.03(0.02)	0.01(0.01)	0.02^***^ (0.01)	0.07^***^ (0.01)
Subjective social class		0.06^***^ (0.02)		0.10^***^(0.02)	0.12^***^(0.03)	0.03^***^(0.01)	0.04^***^ (0.01)	0.11^***^ (0.02)
Social trust		0.02 (0.03)		0.01 (0.03)	0.07 (0.06)	0.01 (0.01)	0.01 (0.02)	0.08^***^ (0.03)
Social net		0.03 (0.02)		0.03 (0.02)	0.07^**^ (0.03)	0.02^**^ (0.01)	0.02^*^ (0.01)	0.03 (0.02)
Constant	−1.39^***^ (0.55)	−1.33^***^ (0.28)	0.76^***^ (0.06)	0.43^*^(0.25)	−4.06^***^(0.49)	0.03(0.10)	−0.80^***^ (0.17)	2.29^**^ (0.27)
Sample	5,101	5,101	5,101	5,101	5,101	5,101	5,101	5,101

*p < 0.1, **p < 0.05, ***p < 0.01. Values in parentheses are standard errors.

### 4.2 Robustness test

We conduct a robustness test by replacing the dependent variable. Charitable donations require individuals to invest their own resources, such as money and time (Costello and Malkoc, 2022). Volunteering is another useful indicator of an individual’s charitable giving behavior. In this study, we use the question from CGSS2012: “In 2011, did you personally participate in any social volunteer activities?” as a substitute for the charitable donation participation variable. A “Yes” answer is coded as 1, and “No” as 0. The question, “In 2011, which of the following areas did you participate in social volunteer activities? How many hours in total did you spend on volunteer work in each area?” is used as a substitute for the donation amount variable. The total amount of an individual’s time donations was calculated by summing their volunteer time across various fields. To reduce data volatility, a logarithmic transformation is applied to the variable. The regression results in Model 5 of Table 2 indicate that physical exercise is a significant positive predictor of volunteer participation, with a regression coefficient of 0.23, significant at the 1% level. Meanwhile, the results in Model 6 of Table 2 suggest that physical exercise also positively influences the duration of volunteer service, with a regression coefficient is 0.06, which is significant at 1% level. These findings further support hypothesis H1.

A robustness test is then performed by altering the regression model. The regression models with donation participation and donation amount as the dependent variables are re-estimated using the Probit and Tobit models, respectively, with the results shown in Models 7 and 8 of Table 2. The regression results indicate that after controlling for demographic characteristics, physical exercise participation associate with both charitable donation participation and donation amount, with effect values of 0.12 and 0.11, respectively, and both are significant at the 1% level.

Since the self-selection issue is not accounted for in the ordered logit and OLS models, selection bias may arise. In this study, a robustness test using propensity score matching (PSM) (Becker and Ichino, 2002) is applied to control for differences in the characteristics of physical exercisers and non-exercisers. This study categorizes “several times a year or less” as irregular physical exercise, which is effectively considered as “no participation.” In summary, the study combines “every day,” “several times a week” and “several times a month” under the label “participation in physical exercise,” which is assigned the code “1.” Conversely, “several times a year or less” and “never” are grouped together under “no participation,” coded as “0.” By estimating propensity scores, the treatment group and the control group are matched to ensure that they have similar distributions across covariates. After matching, a balance test is conducted on the matched samples to confirm that there are no significant systematic differences in the explanatory variables between those who participate in physical exercise and those who do not. The results of the balance test are provided in the Supplementary materials. Based on the matching effect test, the ATT values estimated using the nearest neighbor matching and radius matching methods are 0.11, 0.10 and 0.62, 0.58, respectively, both significant at the 1% level (Table 3). This indicates that even after correcting for sample selection bias, engagement in physical exercise remains positively associated with charitable donation behavior.

**Table 3:** Matching estimation results of propensity score.

Dependent Variable	Matching method	Samples	Treatment group	Control group	ATT	Standard deviation
Donation Participation	Nearest neighbor match	U	0.47	0.27	0.20^***^	0.01
		M	0.47	0.36	0.11^***^	0.02
	Radius match	U	0.47	0.27	0.20^***^	0.01
		M	0.47	0.37	0.10^***^	−0.03
Donation Amount	Nearest neighbor match	U	2.48	1.27	1.21^***^	0.07
		M	2.48	1.87	0.62^***^	0.11
	Radius match	U	2.48	1.27	1.21^***^	0.07
		M	2.48	1.90	0.58^***^	−0.13

***p < 0.01. “M” means the sample was matched, “U” means the sample was not matched.

### 4.3 Heterogeneity analysis

The heterogeneity test results based on gender, region, and age are shown in Table 4. From a gender perspective, participation in physical exercise is positively associated with both male and female charitable donation participation and donation amounts. Regarding household registration type, the results indicate stronger positive associations between physical activity and charitable donation behavior among rural residents compared to their urban counterparts. This may be attributed to the fact that urban residents in China have greater access to a variety of sports activities, resulting in a relatively weaker triggering effect on donation behavior. In contrast, rural residents have fewer opportunities for physical activities, so each instance of exercise has a stronger psychological and emotional impact, making it more likely to stimulate their willingness to donate. Finally, across age groups, physical exercise shows a positive association with charitable donation behavior, with the strongest association observed among individuals under the age of 30. One possible reason is that, the influence of the internet and social media is substantial for young people under 30. Since the Ministry of Civil Affairs designated online charity fundraising platforms in 2016, many charitable appeals are posted on these platforms, attracting greater participation from young people (Tsai and Wang, 2019). As a result, younger individuals may be more exposed to donation opportunities, possibly contributing to the observed stronger association in this group.

**Table 4:** Results of heterogeneity analysis.

Variable		Gender		Household registration		Age		
		Male	Female	Urban	Rural	Younger than 30	Aged 30–59	60 and older
DP	Physical exercise	0.05^***^ (0.01)	0.03^***^(0.01)	0.03^***^ (0.07)	0.05^***^ (0.01)	0.05^***^ (0.07)	0.04^***^ (0.01)	0.01^***^ (0.03)
	Control variable	Yes	Yes	Yes	Yes	Yes	Yes	Yes
	Constant	−0.23^***^ (0.28)	−0.18^***^(0.08)	0.14 (0.09)	0.39^***^ (0.07)	0.55^***^ (0.22)	0.22^***^ (0.09)	0.13^**^ (0.14)
	Sample	2,628	2,473	2,424	2,677	651	3,000	1,450
DA	Physical exercise	0.27^***^ (0.04)	0.21^***^(0.04)	0.19^***^ (0.04)	0.30^***^ (0.04)	0.35^***^ (0.08)	0.25^***^ (0.04)	0.19^***^ (0.04)
	Control variable	Yes	Yes	Yes	Yes	Yes	Yes	Yes
	Constant	0.73^***^ (0.42)	−0.02^***^(0.34)	0.02^***^ (0.49)	1.34^***^ (0.33)	1.28^***^ (1.14)	0.30^***^ (0.44)	0.38^***^ (0.70)
	Sample	2,628	2,473	2,424	2,677	651	3,000	1,450

*p < 0.1, **p < 0.05, ***p < 0.01. The values in parentheses represent the standard errors.

## 5 Mechanisms underlying the relationship between physical exercise and charitable donation behavior

Based on the above theoretical framework and model (Equations 2–3), potential mediating pathways linking physical exercise to charitable donation behavior are examined using AMOS 28.0. Additionally, Post-hoc exploratory analyses are conducted to investigate whether social trust and social network may also function as alternative mediators in the relationship between physical exercise and charitable donation behavior.

Firstly, the mediating path through which physical exercise influence donation participation is examined. The modified model fitting index are as follows: GFI = 0.93; NFI = 0.92; CFI = 1.00; RMSEA = 0.08, which suggest the model is acceptable (Preacher et al., 2011). Results indicate that physical exercise positively associate with both social responsibility (*β* = 0.09, SE = 0.02, *p* = 0.01, 95% CI [0.06, 0.12]) and subjective well-being (*β* = 0.12, SE = 0.01, *p* < 0.01, 95% CI [0.09, 0.14]). Furthermore, social responsibility (*β* = 0.11, SE = 0.01, *p* < 0.001, 95% CI [0.08, 0.13]) and subjective well-being (*β* = 0.01, SE = 0.01, *p* < 0.001, 95% CI [0.06, 0.11]) are found to link positively with donation participation. Importantly, mediation analyses reveal that the indirect effects of social responsibility (*β* = 0.01, SE = 0.00, *p* < 0.01, 95% CI [0.01, 0.02]) and subjective well-being (*β* = 0.01, SE = 0.00, *p* < 0.01, 95% CI [0.01, 0.02]) are statistically significant, as their confidence intervals did not include 0 (see Table 5).

**Table 5:** Bootstrap analysis of mediation effects.

Path	β	BootSE	*p*	95% CI	Relative effect
Total indirect effect	0.02	0.00	***	[0.02, 0.03]	8.34%
Physical exercise → SSR → DP	0.01	0.01	***	[0.01, 0.02]	4.17%
Physical exercise → SWB → DP	0.01	0.00	***	[0.01, 0.02]	4.17%
Direct effect	0.22	0.00	***	[0.34, 0.43]	
Total effect	0.24	0.01	***	[0.22, 0.27]	
Total indirect effect	0.02	0.00	***	[0.02, 0.03]	9.52%
Physical exercise → SSR → DA	0.01	0.00	***	[0.01, 0.02]	4.76%
Physical exercise → SWB → DA	0.01	0.00	***	[0.01, 0.02]	4.76%
Direct effect	0.19	0.01	***	[0.17, 0.22]	
Total effect	0.21	0.01	***	[0.19, 0.24]	

“DP” refers to donation participation, “DA” refers to donation amount, “SSR” refers to responsibility, and “SWB” refers to subjective well-being. *p < 0.1, **p < 0.05, ***p < 0.01. The values in parentheses represent the standard errors.

Secondly, the analysis explores whether social responsibility and subjective well-being serve as potential mediators in the association between physical exercise and donation amount. The revised model’s fitting results are as follows: GFI = 0.93; NFI = 0.94; CFI = 0.94; RMSEA = 0.08. The model is acceptable (Preacher et al., 2011). The model analysis results show that social responsibility (*β* = 0.11, SE = 0.01, *p* < 0.01, 95% CI [0.09, 0.13]) and subjective well-being (*β* = 0.09, SE = 0.01, *p* = 0.011, 95% CI [0.06, 0.11]) positively associate with donation amount, respectively. Moreover, the results confirm that the indirect effects of social responsibility (*β* = 0.01, SE = 0.00, *p* < 0.01, 95% CI [0.01, 0.02]) and subjective well-being (*β* = 0.01, SE = 0.00, *p* < 0.01, 95% CI [0.01, 0.02]) are both statistically significant (see Table 5).

Meanwhile, physical exercise can facilitate the development of individual social capital (Brown et al., 2014; Zhang et al., 2019). As key components of social capital, social trust and social network may influence individuals charitable behavior (Wu et al., 2018). Therefore, they could also serve as mediating factors in the relationship between physical exercise and donation behavior. Consequently, a post-hoc analysis is conducted to explore their mediating mechanism. The results show that the confidence intervals for the mediating effect tests of social trust (95% CI [−0.01, 0.02]) and social network (95%CI [−0.02, 0.02])between physical exercise and donation participation both include 0. Likewise, the confidence intervals for mediating effects of social trust (95% CI [−0.01, 0.01]) and social networks (95%CI [−0.01, 0.01]) on the relationship between physical exercise and donation amount also include zero, indicating that these mediating effects are insignificant.

## 6 Discussion

This study utilizes data from the China General Social Survey (CGSS2012) to examine the relationship between physical exercise and charitable donation behavior among Chinese residents. By considering both altruistic and reciprocal motivations, the research provides a comprehensive analysis of how physical exercise participation is associated with donation participation and donation amounts, as well as the potential pathways that may underlie these associations. The results suggest that physical exercise is positively associated with both donation participation and the amount donated, with social responsibility and subjective well-being serving as possible mediating variables in these relationships.

In contrast, the mediating effects of social trust and social network are found to be statistically insignificant. Previous research has shown that different types of social capital may exert varying influences on charitable behavior (Glanville et al., 2016). As core components of social capital, social trust and social network are inherently multidimensional constructs (Bhandari and Yasunobu, 2009). However, in the CGSS2012 dataset, the measurement of these variables is relatively coarse, lacking differentiation between types of social network and social trust. This measurement limitation may be a potential reason why the mediating roles of social trust and social network are found to be insignificant in this study. Future research would benefit from adopting more refined and multidimensional instruments to better capture the potential mediating roles of social trust and social network linking physical activity and donation behavior.

### 6.1 Theoretical implications

Existing literature has explored the effects of physical exercise from both individual and societal perspectives, highlighting its positive impact on quality of life (Morimoto et al., 2006), interpersonal networks (Shaffer et al., 2024), and community cohesion (Cradock et al., 2009). Ishihara et al. (2023) revealed the neural mechanism of physical exercise stimulating prosocial behavior through EEG analysis. Previous studies have primarily examined the effects of sports on physical health, mental well-being, and social interactions, while relatively little attention has been given to the role of sports in fostering charitable donation behavior. Through empirical analysis, this study is the first to explore the impact of sports participation on charitable giving among Chinese people. Base on the altruistic and reciprocal motives of charitable behavior, this research further investigates the mediating mechanisms of social responsibility and subjective well-being in the relationship between sports participation and philanthropic engagement. By bridging this research gap, the study provides new insights into how physical exercise can serve as a catalyst for prosocial behavior, offering valuable implications for both academic discourse and practical policy initiatives aimed at promoting charitable giving. This study integrates the theories of sports sociology and charitable behavior to explore the relationship between physical exercise and charitable donations. This interdisciplinary perspective not only broadens the research horizon of physical exercise but also offers a novel approach to fostering charitable behavior. Specifically, it highlights how sports can serve as a means to enhance social responsibility and subjective well-being, thereby encouraging individuals to engage in charitable giving.

Physical exercise, as an important social activity, not only improves individuals’ physical and mental health but also enhances their sense of social responsibility and subjective well-being. These traits are closely linked to altruism and reciprocal motivations in charitable donation behavior. Social responsibility, a key predictor of altruistic behavior, encompasses individuals’ concern for organizational interests and the welfare of others (Fischer, 2004). Physical activities not only improve physical fitness and quality of life but also play a crucial role in cultivating responsibility. Hellison (1985) suggested that sports activities can help students develop personal qualities, nurture a sense of social responsibility, and improve problem-solving skills, enabling them to become more responsible and actively engaged citizens. Perks (2007) found that youth participation in sports is positively associated with community involvement in adulthood, with its influence persisting throughout the life course. On the other hand, participation in physical exercise significantly enhances individuals’ subjective well-being. Existing literature has examined the positive role of physical exercise in improving well-being from both physiological (Riddick and Daniel, 1984) and psychological (Edwards, 2006) perspectives. Happier individuals tend to engage in more acts of giving and altruistic behavior. Kushlev et al. (2022) conducted an empirical analysis using data from 163 countries to examine the relationship between subjective well-being and prosocial behavior. Their findings suggest that higher levels of life satisfaction and positive emotions significantly predict an increased likelihood of making monetary donations, volunteering, and helping strangers. The results of this study are consistent with the conclusions of the previous literature. Social responsibility and subjective well-being serve as important bridges between physical exercise and charitable donation behavior, demonstrating significant mediating effects. This study provides new evidence for the positive social functions of physical exercise, validating the benefits of promoting mass sports from the perspective of charitable donations.

### 6.2 Practical implications

Community is the basic unit of social life and serve as critical contextual resources for individuals to engage in physical exercise (Sui et al., 2023). By ensuring sufficient facilities and spaces for community sports and fitness activities, community organizations can not only encourage physical activity but also enhance community cohesion, further fostering a sense of social responsibility. Developing a higher-level public fitness service system with a focus on improving residents’ life satisfaction and well-being is also essential. Physical exercise helps promote charitable donations among residents in China and increases the donation amounts. The government should further establish a synergy mechanism between mass sports and the goal of common prosperity, actively explore pathways to achieve common prosperity through sports, build and improve public service systems for mass sports, and actively advocate for mass sports participation. Promoting mass sports can contribute to the goal of common prosperity, helping to foster individuals’ charitable behavior. Additionally, when designing relevant sports activities, it is essential to consider gender, urban–rural, and age differences to maximize the synergistic effect between sports and charity. The government and relevant social organizations can adopt more targeted strategies to promote sports activities, maximizing the role of physical exercise in advancing social welfare initiatives.

### 6.3 Limitations

Although the direct use of CGSS data can make the survey sample accurately reflect the entire population, this study still has certain limitations due to the constraints of secondary data indicators. To start with, due to objective conditions, a single item was used to measure social responsibility and subjective well-being. This approach may have hindered the comprehensive and accurate capture of these constructs, potentially leading to an underestimation of their mediating effects. To address this issue, future research may employ validated composite scales to independently measure subjective well-being and social responsibility, thereby yielding more precise and reliable research conclusions.

Secondly, the CGSS2012 dataset only provides the information about the physical exercise frequency, without distinguishing different types of activities. Moreover, the answers are based on the respondents’ self-reports, so there may be recall bias and may weaken our ability to fully capture its social dimensions. Future research can combine objective data such as the duration of exercise recorded by wearable devices and more fine-grained activity types (such as distinguishing team sports from individual sports) to examine the impact of physical exercise on donation behavior more comprehensively.

Finally, the core variables in this study were only available from the 2012 CGSS dataset, resulting in a limited number of variables and a relatively low proportion of mediating effects. Although alternative explanations such as social trust and social network are excluded, other potential factors such as personality traits (e.g., extraversion) or community cohesion may still exist. Moreover, as the analysis is based on cross-sectional data, causal relationships between physical exercise and charitable donation behavior cannot be inferred. Future research could utilize more recent longitudinal panel data or experimental designs to incorporate additional variables, further explore other mediating pathways, and more rigorously examine the causal relationship between physical exercise and donation behavior.

## Data Availability

The original contributions presented in the study are included in the article/Supplementary material, further inquiries can be directed to the corresponding author.
