# Supplementary material for: Enhancing generosity through movement: association between physical exercise and charitable donation behavior
Source: Front Psychol. 2025 Jul 22;16:1606795. doi: 10.3389/fpsyg.2025.1606795 (PMC12321790; doi:10.3389/fpsyg.2025.1606795)
Supplement: Supplementary file 1 [file Table_1.doc]

| Variable | Match Type | Treatment Group | Control Group | Standardized Bias(%) | Standardized Bias Reduction（%） | T Value | *p* |
| --- | --- | --- | --- | --- | --- | --- | --- |
| Gender | U | 0.545 | 0.502 | 8.6 | 74.8 | 2.841 | 0.005*** |
|  | M | 0.545 | 0.534 | 2.2 |  | 0.608 | 0.543 |
| Age | U | 48.369 | 49.687 | 8.1 | 63.8 | -2.703 | 0.007*** |
|  | M | 48.369 | 48.862 | 2.9 |  | -0.821 | 0.411 |
| Education | U | 11.014 | 7.761 | 75.9 | 98.1 | 24.481 | 0.000*** |
|  | M | 11.014 | 11.073 | 1.5 |  | -0.414 | 0.679 |
| Marital status | U | 0.777 | 0.812 | 8.9 | 69.3 | -2.963 | 0.003*** |
|  | M | 0.777 | 0.765 | 2.7 |  | 0.764 | 0.445 |
| Party membership | U | 0.21 | 0.088 | 34.8 | 94.7 | 12.342 | 0.000*** |
|  | M | 0.21 | 0.218 | 1.9 |  | -0.522 | 0.602 |
| Ethnicity | U | 0.939 | 0.897 | 15.3 | 74.8 | 4.82 | 0.000*** |
|  | M | 0.939 | 0.929 | 3.8 |  | 1.079 | 0.281 |
| Household registration | U | 0.723 | 0.365 | 77.0 | 96.9 | 25.053 | 0.000*** |
|  | M | 0.723 | 0.712 | 2.4 |  | 0.674 | 0.501 |
| Self-rated health status | U | 3.689 | 3.444 | 23.2 | 82.8 | 7.491 | 0.000*** |
|  | M | 3.689 | 3.649 | 4.0 |  | 1.119 | 0.263 |
| Income | U | 9.145 | 8.4 | 26.0 | 87.5 | 8.627 | 0.000*** |
|  | M | 9.145 | 9.053 | 3.3 |  | 0.915 | 0.360 |
| Subjective social class | U | 4.449 | 4.045 | 24.2 | 84.8 | 7.936 | 0.000*** |
|  | M | 4.449 | 4.388 | 3.7 |  | 1.029 | 0.304 |
| Social trust | U | 3.482 | 3.491 | 0.9 | -48.3 | -0.29 | 0.772 |
|  | M | 3.482 | 3.468 | 1.3 |  | 0.365 | 0.715 |
| Social network | U | 4.241 | 4.159 | 4.8 | 98.1 | 1.553 | 0.121 |
|  | M | 4.241 | 4.242 | 0.1 |  | -0.026 | 0.979 |

**Balance test before and after propensity value matching(Nearest neighbor matching method)**

**p* < 0.1，***p* < 0.05，****p* < 0.01

**Balance test before and after propensity value matching(Radius matching method)**

| Variable | Match type | Treatment Group | Control Group | Standardized Bias(%) | Standardized Bias Reduction（%） | T Value | *p* |
| --- | --- | --- | --- | --- | --- | --- | --- |
| Gender | U | 0.545 | 0.502 | 8.6 | 92.9 | 2.841 | 0.005*** |
|  | M | 0.544 | 0.542 | 0.6 |  | 0.172 | 0.864 |
| Age | U | 48.369 | 49.687 | 8.1 | 90.0 | -2.703 | 0.007*** |
|  | M | 48.358 | 48.459 | 0.8 |  | -0.226 | 0.821 |
| Education | U | 11.014 | 7.761 | 75.9 | 99.3 | 24.481 | 0.000*** |
|  | M | 10.985 | 11.003 | 0.5 |  | -0.145 | 0.885 |
| Marital status | U | 0.777 | 0.812 | 8.9 | 96.0 | -2.963 | 0.003*** |
|  | M | 0.777 | 0.776 | 0.4 |  | 0.099 | 0.921 |
| Party membership | U | 0.21 | 0.088 | 34.8 | 99.2 | 12.342 | 0.000*** |
|  | M | 0.207 | 0.208 | 0.3 |  | -0.079 | 0.937 |
| Ethnicity | U | 0.939 | 0.897 | 15.3 | 90.0 | 4.82 | 0.000*** |
|  | M | 0.939 | 0.936 | 1.5 |  | 0.427 | 0.670 |
| Household registration | U | 0.723 | 0.365 | 77.0 | 99.0 | 25.053 | 0.000*** |
|  | M | 0.722 | 0.725 | 0.7 |  | -0.209 | 0.834 |
| Self-rated health status | U | 3.689 | 3.444 | 23.2 | 93.8 | 7.491 | 0.000*** |
|  | M | 3.689 | 3.679 | 1.4 |  | 0.403 | 0.687 |
| Income | U | 9.145 | 8.4 | 26.0 | 91.7 | 8.627 | 0.000*** |
|  | M | 9.137 | 9.184 | 2.2 |  | -0.607 | 0.544 |
| Subjective social class | U | 4.449 | 4.045 | 24.2 | 93.7 | 7.936 | 0.000*** |
|  | M | 4.441 | 4.423 | 1.5 |  | 0.428 | 0.668 |
| Social trust | U | 3.482 | 3.491 | 0.9 | -181.0 | -0.29 | 0.772 |
|  | M | 3.479 | 3.461 | 2.5 |  | 0.691 | 0.490 |
| Social network | U | 4.241 | 4.159 | 4.8 | 21.8 | 1.553 | 0.121 |
|  | M | 4.234 | 4.189 | 3.7 |  | 1.042 | 0.298 |

**p* < 0.1，***p* < 0.05，****p* < 0.01
